# Autophagy coordinates chondrocyte development and early joint formation in zebrafish

**DOI:** 10.1096/fj.202101167R

**Published:** 2021-10-28

**Authors:** Joanna J. Moss, Martina Wirth, Sharon A. Tooze, Jon D. Lane, Chrissy L. Hammond

**Affiliations:** ^1^ School of Biochemistry University of Bristol Bristol UK; ^2^ School of Physiology, Pharmacology and Neuroscience University of Bristol Bristol UK; ^3^ Molecular Cell Biology of Autophagy The Francis Crick Institute London UK; ^4^ Present address: Department of Cell and Chemical Biology Leiden University Medical Center Leiden The Netherlands

**Keywords:** Atg13, autophagy, chondrocytes, joints, zebrafish

## Abstract

Autophagy is a catabolic process responsible for the removal of waste and damaged cellular components by lysosomal degradation. It plays a key role in fundamental cell processes, including ER stress mitigation, control of cell metabolism, and cell differentiation and proliferation, all of which are essential for cartilage cell (chondrocyte) development and survival, and for the formation of cartilage. Correspondingly, autophagy dysregulation has been implicated in several skeletal disorders such as osteoarthritis and osteoporosis. To test the requirement for autophagy during skeletal development in zebrafish, we generated an *atg13* CRISPR knockout zebrafish line. This line showed a complete loss of *atg13* expression, and restricted autophagic activity in vivo. In the absence of autophagy, chondrocyte maturation was accelerated, with chondrocytes exhibiting signs of premature hypertrophy. Focussing on the jaw element, autophagy disruption affected joint articulation causing restricted mouth opening. This gross behavioural phenotype corresponded with a failure to thrive, and death in homozygote *atg13* nulls within 17 days. Taken together, our results are consistent with autophagy contributing to the timely regulation of chondrocyte maturation and for extracellular matrix formation.

**Abbreviations**
ATG: autophagy‐related
BafA1: Bafilomycin A1
Col10a1: type X collagen α1
Col2a1: type II collagen α1
dpf: days post fertilization
ECM: extracellular matrix
EM: electron microscopy
HORMA: Hop1/Rev7/Mad2
hpf: hours post fertilization
LC3: MAP1LC3
OA: osteoarthritis
PI3KC3: phosphatidyl inositol 3‐kinase complex I
ULK: Unc‐51‐like autophagy‐activating kinase

## 1 INTRODUCTION

Macroautophagy (henceforth termed autophagy) is a catabolic process which enables the breakdown of cytosolic components—including misfolded protein aggregates, redundant organelles, and invading microorganisms—into their basic biomolecular constituents by the actions of lysosomal acid hydrolases. This cellular recycling pathway is essential during differentiation, and contributes to cell and tissue homeostasis, where its primary function is to mobilize nutrients to sustain vital cellular functions during stress.^1^ It involves the formation of an isolation membrane that sequesters cytoplasmic cargo, and this membrane structure finally seals to form a new organelle—the autophagosome—which is subsequently delivered to the lysosome for degradation. This process is mediated by a collection of autophagy‐related (ATG) proteins which can be categorized into four functional complexes: the Unc‐51‐like autophagy‐activating kinase (ULK) complex; the phosphatidyl inositol 3‐kinase complex I (PI3KC3); and the two conjugation systems, ATG5‐ATG12‐ATG16L and MAP1LC3‐ATG3.^2^, ^3^

In vertebrates, autophagy initiation requires the formation of the ULK complex, comprising the serine/threonine protein kinase ULK1/2, and its adaptors, ATG13, FIP200, and ATG101.^4^ Upon autophagy induction, the ULK complex translocates to discrete foci dispersed throughout the cell, typically associated with endoplasmic reticulum (ER) membrane, where it phosphorylates components of PI3KC3, triggering recruitment of the remaining core autophagy machinery.^2^, ^5^ Along with FIP200, ATG13 has been shown to play a vital role in both the localized activation of the ULK1 kinase and its recruitment at the nascent isolation membrane site.^6^, ^7^ Here, ATG13 helps form a building scaffold for other proteins within the autophagy pathway to bind and be stabilized.^8^ These roles require the two key domains that comprise ATG13: the C‐terminal Hop1/Rev7/Mad2 (HORMA) domain, and the N‐terminal IDR (intrinsically disordered region).^9^, ^10^ In particular, the highly conserved HORMA domain is essential for autophagy induction and the recruitment of PI3KC3 via ATG14.^11^ In a range of vertebrate models and cell lines, loss of ATG13 expression has been shown to block autophagy activity (^12^, ^13^, ^14^, ^15^, ^16^, ^17^; see table 3 in Ref. [18]). Meanwhile, mouse knockout models for Atg13 are embryonic lethal, a result consistent with the targeting of other autophagy genes in murine knockout models, including Fip200, and Atg7.^19^, ^20^, ^21^ Interestingly, mouse knockout models have demonstrated that, generally, loss of upstream autophagy genes causes lethality earlier in development compared to *atg* genes encoding members of the conjugation systems: Beclin1 and Vps34 (in addition to those mentioned above) are embryonically lethal,^22^, ^23^ whereas genes acting later (Atg3, Atg5, Atg7, Atg9a, Atg12, Atg16L1 and by contrast, Ulk1/2) show neonatal lethality (see table 1 in Ref. [24]). A result also mirrored in knockout zebrafish lines for the equivalent *atg* genes.^25^ Therefore, these results demonstrate that autophagy and its machinery are involved in multiple cellular processes beyond metabolic recycling, and are thus essential for survival.^26^

Extensive studies have established the importance of autophagy in a range of housekeeping pathways, such as the control of cell metabolism, mitigation against endoplasmic reticulum (ER) and mitochondrial stress, in addition to roles during the coordination of cellular differentiation and proliferation.^27^, ^28^, ^29^ Each of these processes have been shown to be essential for cartilage cell (chondrocyte) development and survival, and for chondrogenesis—the formation of cartilage from condensed mesenchymal tissue.^30^, ^31^, ^32^ Correspondingly, autophagy dysregulation has been implicated in several skeletal disorders, including the degenerative joint disorder, osteoarthritis (OA).^32^, ^33^, ^34^, ^35^, ^36^ OA is the most common cause of arthritis in the world^37^, ^38^ and one of the biggest causes of disability, causing an increasing global economic burden through lost working days and forced early retirement.^39^ OA affects all structures of the joint and is characterized by the progressive degeneration of cartilage causing, synovial inflammation, osteophyte formation, ligament damage, bone misalignment, and joint pain.^40^, ^41^ Although often characterized as a disease of ageing, recent studies have highlighted the effect of improper joint shape formation during skeletogenesis on OA development in later life.^42^, ^43^, ^44^, ^45^ Therefore, determining the impact of cellular processes, such as autophagy, on the co‐ordination of cartilage and joint development is essential for deepening our understanding of the pathogenesis of OA and how this disease can be best treated.

The process of chondrogenesis begins with the condensation and differentiation of mesenchymal stem cells at sites of future skeletal formation. These cells differentiate into cartilage forming chondrocytes which secrete a characteristic extracellular matrix (ECM) formed largely of type II collagen α1 (Col2a1) and specific proteoglycans, such as aggrecan, generating a cartilage matrix.^46^ As chondrocytes develop, they pass through a well characterized set of maturation steps which include intercalation and proliferation, where cells flatten and separate into a narrow, single‐cell stacked column, followed by hypertrophication, as cells exit the cell cycle and switch from secreting Col2a1 to type X collagen α1 (Col10a1).^47^, ^48^, ^49^

Several studies have identified important roles for autophagy throughout chondrogenesis. In the early stages of this process, in vitro studies have shown a positive correlation between autophagy activity and chondrocyte proliferation and differentiation,^50^ whilst maturing chondrocytes show high MAP1LC3 expression.^51^ In mice, during early chondrogenesis, chondrocyte autophagy is induced in the growth plate postnatally,^52^ and the conditional loss of Atg5 or Atg7 during chondrogenesis has been shown to reduce growth plate activity and cause growth retardation,^50^ which is likely triggered by ER stress within chondrocytes.^53^ Additionally, conditional knockout of Atg7 in mouse chondrocytes causes enlarged ER cisternae, ECM disorganization and retention of procollagen 2 (a preform of Col2a1—a major component of cartilage matrix), as well as reduced chondrocyte proliferation and survival.^52^, ^53^ If autophagy is disrupted later in chondrogenesis, chondrocytes in the proliferative stage show an accumulation of glycogen granules, severe growth retardation, and increased apoptosis.^30^ Meanwhile, looking beyond early development, reduced autophagy activity via a chondrocyte‐specific Atg5 deletion causes the premature onset of OA development in mice from 6 months.^34^ Together these studies demonstrate a key role for autophagy in chondrocyte proliferation and cartilage growth; and highlight how disruption to this process can lead to the premature development of degenerative joint disease. However, the role of the autophagy pathway within chondrocyte differentiation is still incompletely understood.

In this study, we have generated a new *atg13* knockout zebrafish line and characterized its role during cartilage and joint development. We find that suppression of autophagy accelerates chondrocyte maturation, leading to improper chondrocyte intercalation and disruption to jaw joint formation and movement. We argue that this developmental defect contributes to lethality at around day 17, due to a failure of *atg13* knockout fish to thrive at free‐feeding stages. The disruption to joint function seen in this model indicates an important role for autophagy in supporting the regulation of chondrocyte development to ensure proper joint formation and highlights an important pathway through which autophagy dysregulation may contribute to OA development.

## 2 METHODS

### 2.1 Zebrafish husbandry and transgenic lines

Zebrafish were raised and maintained under standard conditions.^54^ Experiments were performed under UK Home Office project licences, under approval by the local ethics committees (the Animal Welfare and Ethical Review Body of the University of Bristol and the Francis Crick Institute). Transgenic lines *Tg(CMV:EGFP‐map1lc3b)* ^55^ and *Tg(Col2a1aBAC:mcherry)* ^56^ have been reported previously.

The *atg13* stable zebrafish line (*atg13^fci500^*) was generated using CRISPR‐Cas9 mutagenesis. Briefly, gRNAs targeting exon 3 of the zebrafish *atg13* orthologue (Ensembl: ENSDART00000052324.6; zgc:63526) were cloned into the pT7‐gRNA plasmid (Addgene #46759) and generated according to Jao et al.^57^; CRISPR1‐atg13 (exon 3)‐For: 5′‐TAGGTTATAGTGCAAGCCCGGCT‐3′ and CRISPR1‐atg13 (exon 3)‐Rev: 5′‐AAACAGCCGGGCTTGCACTATAA‐3′. Following generation of Cas9 mRNA using the pT3TS‐nls‐zCas9‐nls plasmid (Addgene #46757),^57^ Cas9‐encoding mRNA (200 ng/µl) and *atg13* targeting gRNA (~16–25 ng/µl) were co‐injected into one‐cell stage embryos. High‐resolution melting analysis (HRMA)^58^ was performed on DNA from single embryos and fin clips to determine the efficacy of mutagenesis in F0 and F1 embryos and to identify adult F1 heterozygous carriers. The PCR product sequenced and a 5‐base pair (bp) deletion at 92–96 bp (31aa, zv9 chr19:7834334) in the *atg13* gene was detected, causing a premature stop codon and insertion of a novel HindIII restriction site at the mutation site.

For autophagy flux analysis, larvae were treated at 3 or 4 days post fertilization (dpf) with 100 nM Bafilomycin A1 (BafA1; 14005, Cambridge Bioscience, UK) or DMSO diluted in Danieaus for 3 h at 28°C.

### 2.2 LysoTracker live staining

For visualization of lysosomal compartments, LysoTracker Red DND‐99 (L‐7528, Invitrogen, MA, USA) was used. Following treatment with DMSO or BafA1, *Tg(atg13;CMV:EGFP‐map1lc3b)* 4 dpf larvae were placed in 10 µM LysoTracker Red in Danieaus for 1 h at 28°C and then washed three times (3 min per wash) in fresh Danieaus. LysoTracker‐positive and GFP‐Lc3 puncta were counted from single z‐slices from three independent fields of a set size per larvae.

### 2.3 Cell proliferation assay

The proliferation of the larvae was measured using the Click‐iT EdU imaging kit (Invitrogen) according to the manufacturer's instructions. Briefly, *Tg(atg13; Col2a1aBAC:mcherry)* larvae at 5 dpf were treated with 400 μM EdU in Danieaus for 24 h. Larvae were fixed in 4% PFA and then incubated in the Click‐iT reaction cocktail for 30 min.

### 2.4 DNA extraction and genotyping

Whole embryos or caudal fin amputations on larvae^59^ at 3–5 dpf were incubated in base solution (25 mM NaOH, 0.2 mM EDTA) for 30 min at 95°C before addition of equal volume of neutralization solution (40 mM Tris–HCl, pH 5.0). For genotyping, touchdown PCR (with primers *atg13* F‐ GGCTCGTGCGACAATGGATAGTG; R‐ GACCTCGGGGATGTCCTTTATTGC) was followed by a HindIII restriction digest (R3104S, New England Biolabs, MA, USA), and fragments were separated by gel electrophoresis on a 3% agarose gel. Digestion product for wildtype PCR product is 386 bp; 201 bp for *atg13*‐mutant allele.

### 2.5 Western blotting

Five days post fertilization larvae were deyolked and snap‐frozen in liquid nitrogen. Hot 3× SDS sample buffer (100 µl per 15 larvae at 5 dpf) was added and samples homogenized using a pellet pestle (Z359971, Sigma, MI, USA) for 45 sec and by pulling sample through a 0.22‐gauge needle on ice 6–8 times. Lysates were heated at 95°C for 10 min and resolved on 10% or 12% polyacrylamide gels at 40 mA after loading 25 µl per sample. Following transfer at 100 V, nitrocellulose membranes were blocked in 5% milk‐TBS‐1% Triton (TBS‐T), then incubated with primary antibodies diluted in 5% milk overnight at 4°C (anti‐ATG13 (SAB4200135, 1:100, Sigma); anti‐GAPDH (10494‐1‐AP, 1:2000, Proteintech, IL, USA); anti‐LC3B (ab51520, 1:300, Abcam, Cambridge, MA, USA); anti‐p62/SQSTM1 (5114, 1:300, Cell Signalling Technologies, MA, USA)). After washing in TBS‐T, blots were incubated with secondary anti‐rabbit (G33‐62G, 1:10 000, SignalChem, Canada) and anti‐mouse (G32‐62G, 1:10 000, SignalChem) horseradish peroxidase‐conjugated antibodies at room temperature for 1 h before exposure on photographic film (28906844, Amersham, UK) following incubation with ECL for 1 min (RPN2232, GE Healthcare, IL, USA) as per the manufacturer's instructions.

### 2.6 Whole‐mount immunohistochemistry

Larvae were fixed in 4% PFA then stored at −20°C in 100% methanol. Larvae were re‐hydrated, washed in PBS‐T before permeabilization with proteinase K (4333793, Sigma) at 37°C. For A4.1025 antibody staining, larvae were instead permeabilized in 0.25% Trypsin for 15 min on ice. Larvae were then blocked in 5% horse serum and incubated with primary antibodies overnight at 4°C, unless otherwise stated (anti‐myosin (clone A4.1025) (sc‐53088, 1:500, Insight Biotech, UK); anti‐GFP (ab13970, 1:200, Abcam); anti‐mCherry (M11217, 1:100, Invitrogen); anti‐col2a1 (II‐II6B3‐s, 1:20, DSHB, IA, USA); anti‐colXa1 (SAB4200800, 1:100, 48 h incubation needed, Sigma); anti‐sox9a (GTX128370, 1:300, Genetex, CA, USA)). Larvae were washed extensively before incubation with Alexa‐Fluor secondary antibodies (Invitrogen) diluted in 5% horse serum at 1:500 for 2 h at room temperature in the dark. For DAPI staining, larvae incubated in PBS‐T containing DAPI (1 µg/ml) for 1 h at room temperature before washing.

### 2.7 Confocal microscope imaging of larvae

For confocal imaging, larvae were mounted ventrally in 1% LMP agarose (16520050, Thermofisher, MA, USA) and imaged using a Leica SP5‐II AOBS tandem scanner confocal microscope attached to a Leica DMI 6000 inverted epifluorescence microscope and oil immersion 20× or 40× objectives. The microscope was located in the Wolfson Bioimaging Facility, Bristol and run using Leica LAS AF software (Leica, Germany). Maximum projection images were assembled using LAS AF Lite software (Leica) and Fiji.^60^

### 2.8 Stereomicroscope imaging of zebrafish

Images of live larvae from 1–7 dpf were obtained using a Leica MZ10 F modular stereo microscope system at 1–8.3× magnification. For live imaging, fish were anesthetized using 0.1 mg/ml MS222 (Tricaine methanesulfonate) diluted in Danieaus and imaged laterally.

### 2.9 Measurement of jaw movement frequency

High‐speed movies of 1000 frames were made of jaw movements of *wt* and *atg13* mutants at 5 dpf. The number of mouth movements was recorded per 1000 frames for 5 *wt* and *atg13* mutants, respectively. Frames displaying the three widest jaw openings were selected per fish and the distance between the lower and upper jaw at each joint calculated in µm.

### 2.10 Transmission electron microscopy

Following treatment with DMSO or BafA1 for 3 h, larvae at 5 dpf were fixed in 2.5% glutaraldehyde in 0.1 M sodium cacodylate buffer (pH 7.3) at 4°C, washed and then fixed in 0.2 M osmium in sodium cacodylate buffer with 1.5% ferrocyanide for 1 h at room temperature. After washing, larvae were placed into a sample processor for transmission electron microscopy (TEM) using a standard Epon resin protocol. Briefly, larvae were postfixed in reduced osmium, stained with uranyl acetate dehydrated in ethanol and infiltrated with Epon resin via propylene oxide, and polymerized at 60°C for 48 h.

Ultra‐thin sections (50 nm) of epon embedded larvae were cut using a diamond knife, collected on Formvar coated one‐hole copper grids (AGS162, Agar Scientific, UK) and observed using a Tecnai 12‐FEI 120 kV BioTwin Spirit transmission electron microscope. Images of chondrocytes from transverse sections of the ethmoid plate were collected, (*n* = 3 larvae per genotype, per condition) using an FEI Eagle 4k × 4k CCD camera and analyzed using the freehand selection tool and multi‐point counter in Fiji.^60^

### 2.11 Image analysis and statistics

Larval lengths were obtained from lateral images at 1–7 dpf by measuring from the tip of the mouth to the end of the tail manually in ImageJ.

Whole jaw measurements and muscle fibre number and length measurements were taken manually in ImageJ from max projections of confocal *z*‐stacks of *wt* and *atg13*‐mutant larvae immunostained with Col2a1 or A4.1025 antibodies, respectively.

Modular cell analysis was performed using the freely available Modular Image Analysis (MIA; version 0.9.30) workflow automation plugin for Fiji developed by Dr Stephen Cross.^60^, ^61^ Program requires the input of confocal *z*‐stacks of larval jaw joints, labelled for Col2a1 to allow for the calculation of cell number and volume, jaw element volume and distance, and inter‐element volume.

To quantify Sox9a expression, the Seg3D program was used (a custom script written in MATLAB (version 2015a; Mathworks) developed by Dr Stephen Cross and previously described^62^) whereby the volume of Sox9a positive expression within the Col2a1 positive cells is calculated using confocal *z*‐stacks in specified regions of interest which are selected via a freehand tool within the program. Same threshold value was used for each individual sample and calculated by averaging the mean of the automatic threshold value given for each stack for *wt* and *atg13* mutants, respectively.

Statistical analyses were performed using Graphpad Prism v.9. Error bars on all graphs represent the mean ± standard deviation.

## 3 RESULTS

### 3.1 Establishment and characterization of the *atg13* knockout zebrafish model

**FIGURE 1:**
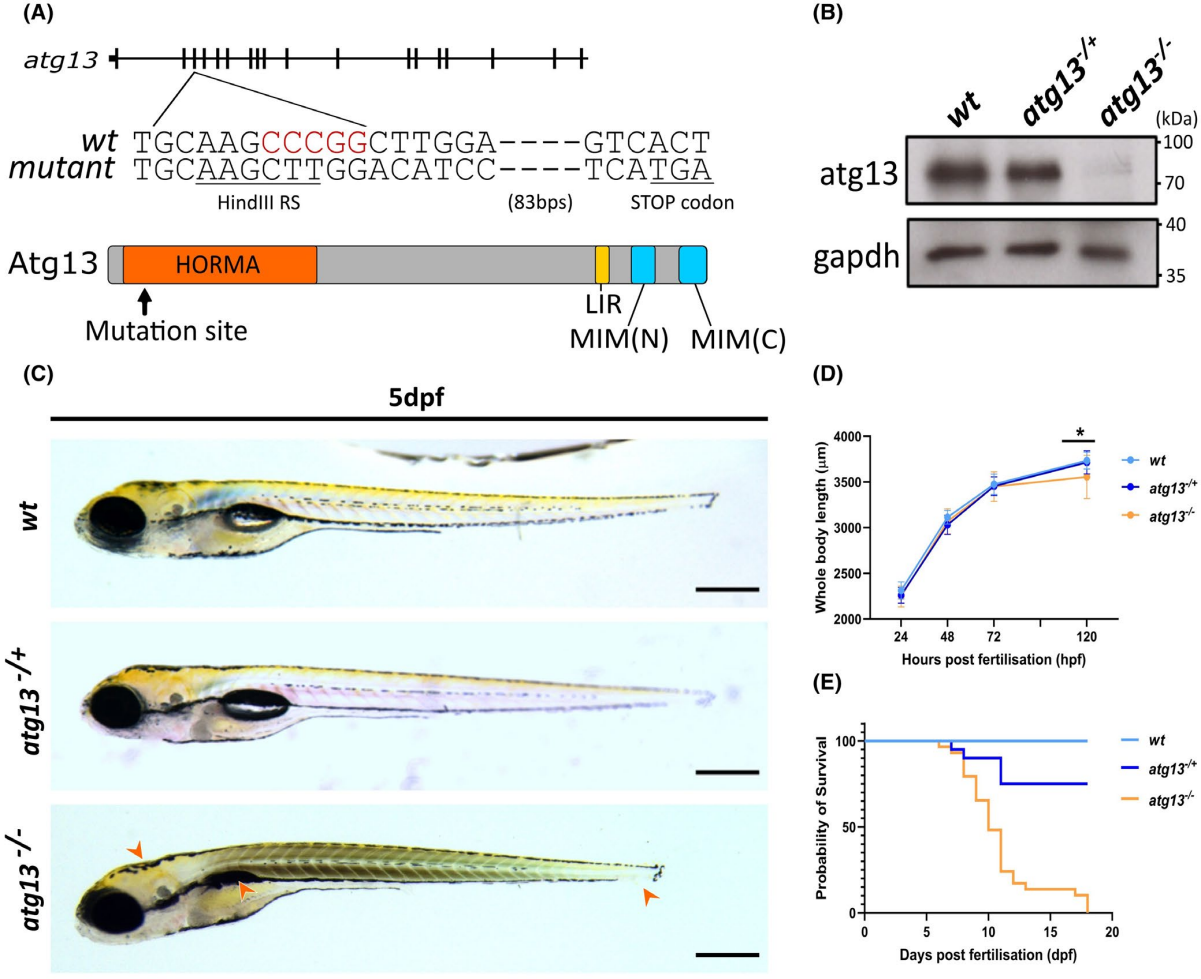
Generation of *atg13* knock out zebrafish line. (A) Schematic showing location of 5 bp deletion in *atg13* knockout line and generation of premature STOP codon, with black lines representing exons. Key domains within atg13 highlighted; LIR, Lc3‐interacting region; MIM, Microtubule interacting motif. (B) Immunoblot showing loss of atg13 expression in *atg13* homozygous mutant. (C) Lateral widefield images of *atg13* zebrafish larvae at 5 dpf. Orange arrowheads indicate phenotypic differences in development between *wt* and *atg13* mutants (from left to right: bent body axis, uninflated swim bladder and shorter body length). Scale bar = 500 µm. (D) Graph showing whole body length of *atg13* larvae from 1–5 dpf. Student's *t*‐test performed at 5 dpf between *wt* and *atg13* mutant, *p = .0013. (E) Kaplan–Meier graph showing survival of larvae up to 20 dpf

To explore the role of autophagy in bone and joint development, we developed an *atg13* knockout zebrafish line using CRISPR‐Cas9 mutagenesis. The line has a 5 bp deletion in exon 3 of *atg13*, resulting in a premature stop codon 97 bp downstream of the mutation site, and the introduction of a diagnostic HindIII restriction site (Figure 1A). The mutation occurs in the highly conserved HORMA domain of Atg13, essential for Atg13 function as a component of the ULK1 complex, and necessary for autophagy initiation.^2^ Accordingly, homozygous *atg13* mutants show complete loss of Atg13 protein expression, while heterozygotes have no discernible difference in protein expression compared to *wt* (Figure 1B), indicative of increased expression from the single remaining allele. These data confirm that the mutation causes a full knockout of Atg13 in the homozygous mutants. Given this, we used the homozygous *atg13* mutants for the majority of experiments in this study, and henceforth, these will be termed “*atg13* mutants.”

At 24 hours post fertilization, *atg13*‐mutant larvae showed no phenotypic differences from *wt*, although by 3 dpf certain developmental differences were observed (bent body axis, oedema; Figure S1). By 5 dpf, *atg13* mutants were significantly shorter in length and displayed at least one phenotypic difference when compared to *wt* and *atg13* heterozygotes, such as edema, bent body axis and a failure to fully use yolk sac (indicated by higher yolk sac mass) or inflate the swim bladder, the latter being indicative of a delay in normal development (Figures 1C,D and S1). In line with other mouse and zebrafish knock‐out models for key ATG genes,^25^, ^63^ *atg13* mutants showed juvenile lethality at 17 dpf (Figure 1E). Taken together, these results suggest that Atg13 is essential for zebrafish survival into adulthood, and that loss of Atg13—although not impacting on early survival—disturbs larval developmental rate and morphology.

### 3.2 *atg13* mutants show reduced autophagy flux

**FIGURE 2:**
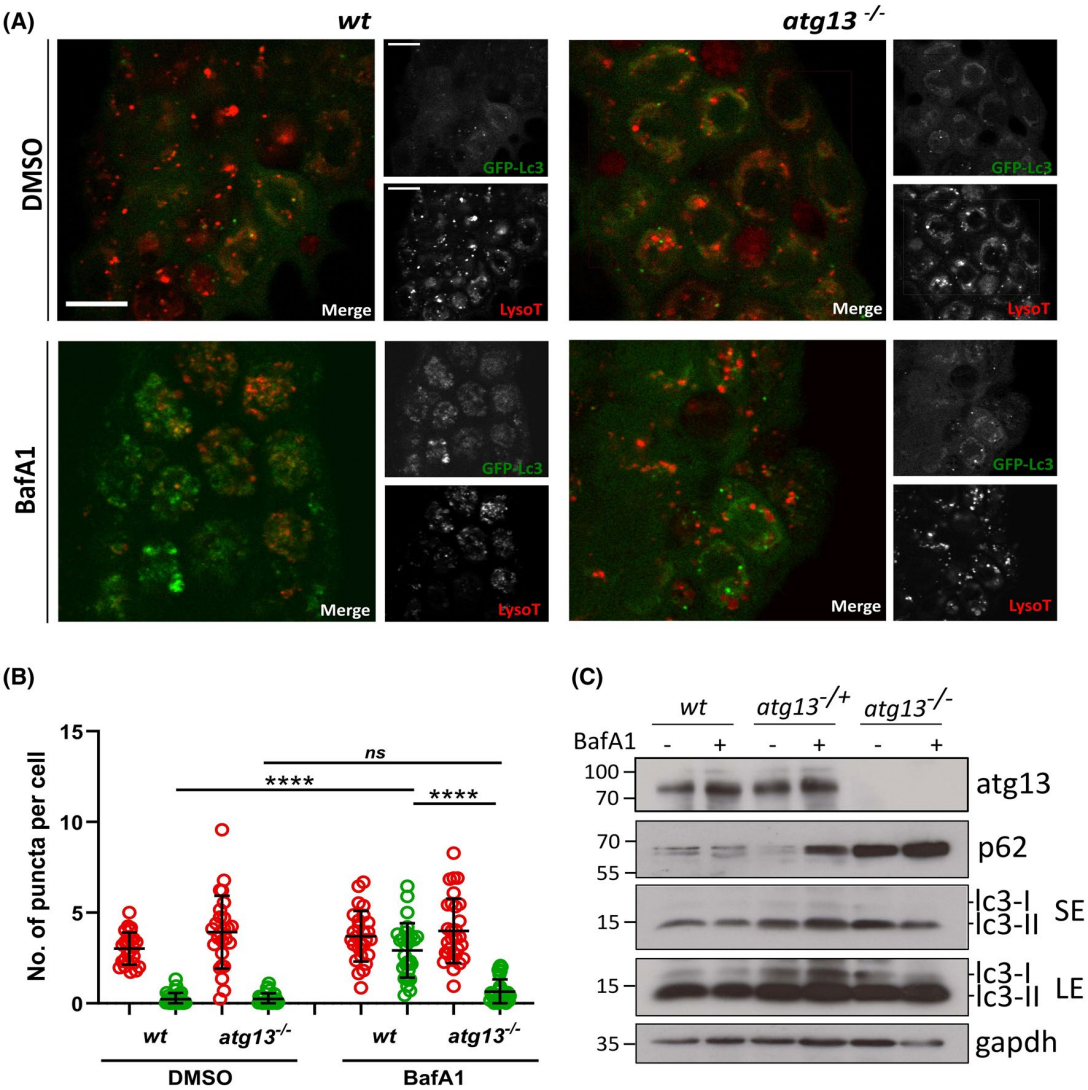
*atg13* mutants show reduced autophagy flux. (A) Representative single confocal z‐slices of epidermal cells taken from LysoTracker stained *Tg(cmv:gfp‐map1lc3;atg13) wt* and mutant larvae, at 4 dpf following treatment with DMSO or 100 nM Bafilomycin for 3 h. Scale bars = 10 µm. (B) Quantification of number of lysosomal (red) and GFP‐Lc3 (green) puncta per cell. Two‐way ANOVA performed for each; ****p < .0001. (C) Representative immunoblot of *atg13 wt*, heterozygous and mutant larvae at 5 dpf following treatment with DMSO or 100 nM Bafilomycin for 3 h. LE, long exposure; SE, short exposure. Molecular weight markers indicated on right hand side of immunoblots

To confirm that autophagy is functionally disrupted in the *atg13* mutants, we generated GFP tagged map1lc3 (GFP‐Lc3) zebrafish^55^ in the *atg13*‐mutant background (*Tg(cmv:gfp‐map1lc3b);atg13)*. Following autophagy initiation, cytosolic Lc3‐I is conjugated to phosphatidylethanolamine, a constituent of the phagophore (or isolation membrane), forming lipidated Lc3‐II which has greater mobility on SDS‐PAGE gels and can be detected in GFP‐Lc3 zebrafish as discrete GFP‐positive puncta using fluorescence microscopy.^64^ By applying LysoTracker Red, a vital lysosomal dye, we measured the abundance of autophagosomes and lysosomes in *wt* and *atg13* mutants at 4 dpf, by counting the number of GFP‐Lc3 and LysoTracker positive puncta per cell (Figure 2A). Under basal conditions (DMSO treatment), *atg13* mutants showed no differences in lysosome abundance compared to *wt*, with a non‐significant decrease in GFP‐Lc3 puncta numbers (Figure 2A,B). Although the loss of *atg13* is expected to prevent autophagosome formation through inhibiting early autophagic signalling,^6^ GFP‐Lc3 positive puncta were present in *atg13* mutants. GFP‐Lc3‐positive aggregates have previously been observed in the mouse *atg13*‐mutant model^20^ and in *C. elegans atg13/epg1*‐mutant embryonic cells^65^, ^66^ and we propose may also occur in our atg13‐mutant zebrafish model.

Next, to analyze the rate of autophagy flux, larvae were treated with the vacuolar‐type H^+^‐ATPase inhibitor, BafA1. As an inhibitor of lysosomal acidification, BafA1 prevents the formation of autolysosomes, and thus causes accumulation of Lc3‐II and autophagic cargo primarily by blocking the degradation of autophagosomes. By comparing Lc3‐II puncta numbers and Lc3‐II density by immunoblotting under basal and BafA1 treated conditions, autophagic flux rates can be assessed. Following treatment with BafA1, *wt* zebrafish showed a significant increase in GFP‐Lc3 puncta numbers compared to basal conditions, indicative of high autophagic flux, as expected (Figure 2A,B). However, there was a dramatically reduced accumulation of GFP‐Lc3 puncta in *atg13* mutants, indicating attenuated autophagic flux (Figure 2A,B).

Immunoblot analysis of endogenous LC3 did not reveal clear differences in the abundance of lipidated LC3‐II between *wt*, heterozygous and homozygous *atg13* mutants under basal conditions and in response to BafA1 treatment (Figure 2C). In contrast to human or rodent samples, zebrafish appear to show higher basal lipidated LC3‐II levels from 2 dpf,^55^, ^67^, ^68^, ^69^ which could be due to differences in LC3 processing, and we observed a similar pattern even in the absence of atg13 expression. More informative, however, was the analysis of the autophagy receptor protein p62 (SQSTM1), which is degraded by the autophagy machinery, and is thus a good measure of flux. We observed a strong accumulation of p62, indicative of defective autophagic flux in the atg13 mutants (Figure 2C). Taken together, these results demonstrate that loss of *atg13* causes the expected deficiencies in autophagy, correlating with a reduced turnover of autophagic substrates. Therefore, under a stress phenotype, *atg13* mutants have a significantly reduced capacity for an autophagy response.

### 3.3 Joint function is reduced in *atg13*‐mutant fish

Given the role of autophagy in key skeletal processes, we examined the effect of the *atg13* mutation on cartilage development in the context of joint formation. In zebrafish, skeletal formation begins as early as 2 dpf with the initial establishment of specific craniofacial cartilaginous structures.^70^ Zebrafish skeletal physiology is comparable to that of mammals, as they share the same joint types and components such as joint cavities, articular cartilage, and synovial membranes.^71^ This has been most extensively shown in the larval zebrafish jaw, which has a synovial joint and is often used to model joint development. Therefore, lower jaw elements in zebrafish larvae were used to compare the development of the cartilaginous skeleton template between *wt* and *atg13* mutants.

**FIGURE 3:**
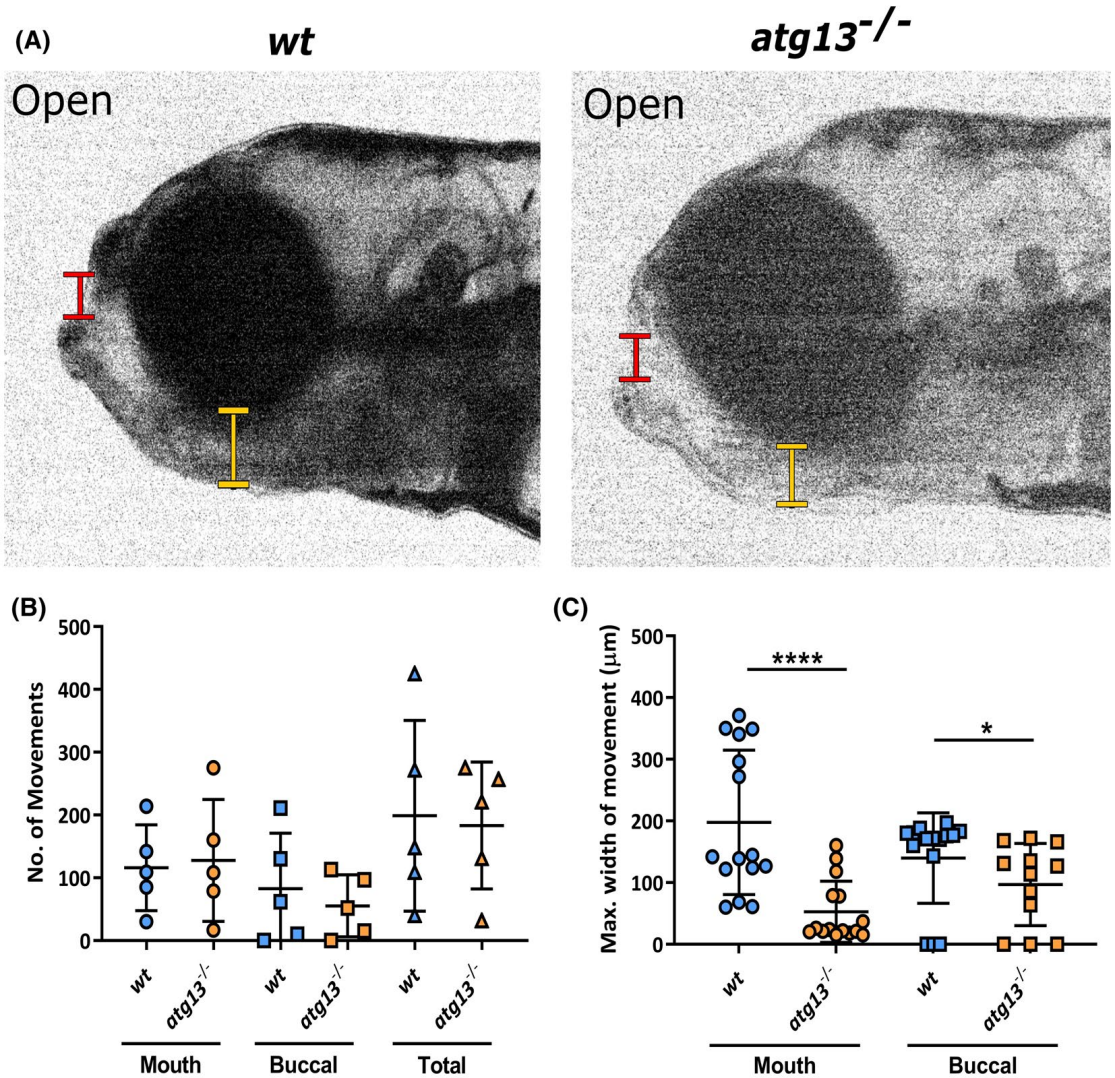
*atg13* mutation reduces jaw function. (A) Stills from videos of larval jaw movements taken at 5 dpf of *wt* and *atg13*‐mutant fish. Red and yellow lines indicate where mouth and buccal width measurements taken from, respectively. Quantification of number (B) and displacement (C) of jaw movements at the mouth and buccal joint. *n* = 5 for each genotype; three widest jaw openings taken per larvae. Student's unpaired *t* test performed for (C), ****p < .0001, *p = .0129

Zebrafish make two distinct jaw movements for feeding and for breathing, which here we describe as mouth and buccal jaw movements, respectively (Figure 3A; red for mouth movements and yellow for buccal movements).^72^ For more detailed analysis, videos of jaw movements were taken at 5 dpf, and the numbers of movements at either joint were measured alongside the displacement distance between the upper and lower jaw (Figure 3; Videos S2 and S3). We observed that the *atg13* mutants did indeed have a significantly reduced range of motion at both joints compared to *wt*, although the total number of movements remained unaffected (Figure 3B,C). The jaw joint itself showed the greatest reduction in movement in *atg13* mutants indicating that these have restricted mouth opening which could impede feeding. We performed whole‐mount immunohistochemistry labelling of myosin in 5 dpf larvae to determine whether the different ranges of jaw movements were caused by defects in the jaw muscle architecture (Figure S4A). Crucially, the *atg13* mutants showed no differences in muscle fibre number compared to *wt* (Figure S4B), whilst the width and length of the intermandibularis posterior and interhyal muscles were also comparable (Figure S4C; width data not shown). These data indicate that the changes to jaw movements are not being caused by defects in muscle patterning but are due to altered joint formation and function.

### 3.4 Reduced chondrocyte proliferation in *atg13* mutants

**FIGURE 4:**
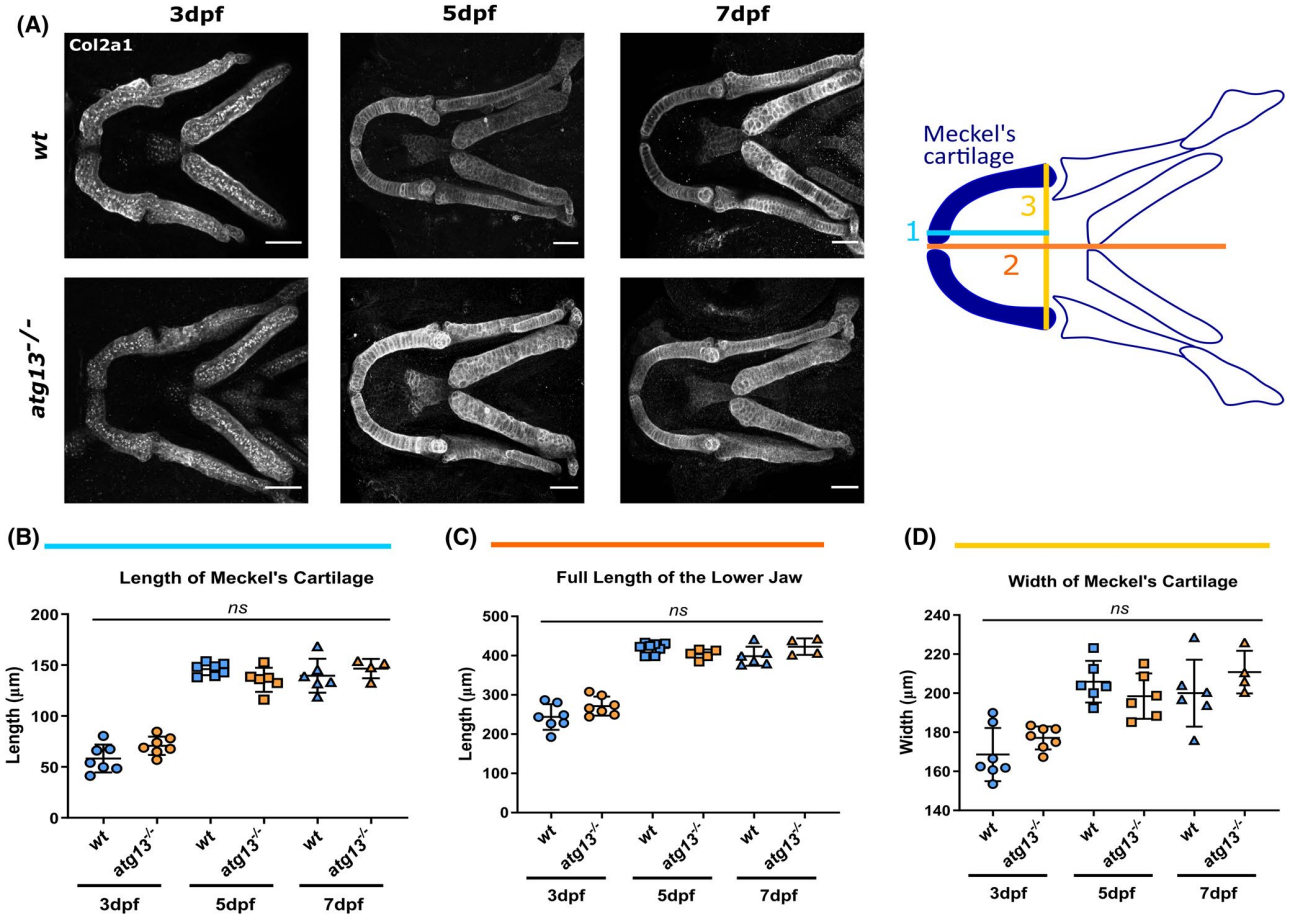
Loss of *atg13* does not affect size of lower jaw in development. (A) *Left*, representative confocal *z*‐stack projections of the lower jaw at 3, 5, and 7 dpf in *wt* and *atg13*‐mutant larvae, immunostained for collagen Type II (Col2a1). Scale bar = 50 µm. *Right*, schematic showing where 3 measurements taken within lower jaw of larvae. (B–D) Quantification of three measurements; (B) length of Merkel's cartilage, (C) length of lower jaw, (D) width of Merkel's cartilage. *n* = 7 for 3 dpf, *n* = 6 for 5 dpf and *n* = 6 and 4 at 7 dpf for *wt* and *atg13* mutant, respectively. Student's unpaired *t* test performed for each age, ^ns^ *p* > .05

Given these changes to jaw opening, we next investigated whether these were due to the loss of autophagy activity affecting the overall growth and formation of the jaw. At 3, 5, and 7 dpf, the length and width of lower jaw dimensions were measured using immunostaining of Col2a1 (Figure 4A). From this analysis, it was evident that no changes in Meckel's cartilage length and width, as well as the full length of the lower jaw were observed between the *wt* and *atg13*‐mutant fish (Figure 4B–D). From this, we inferred that changes to cartilage development caused by the absence of *atg13* appear to be limited to the joint site only and to the cells forming this region.

**FIGURE 5:**
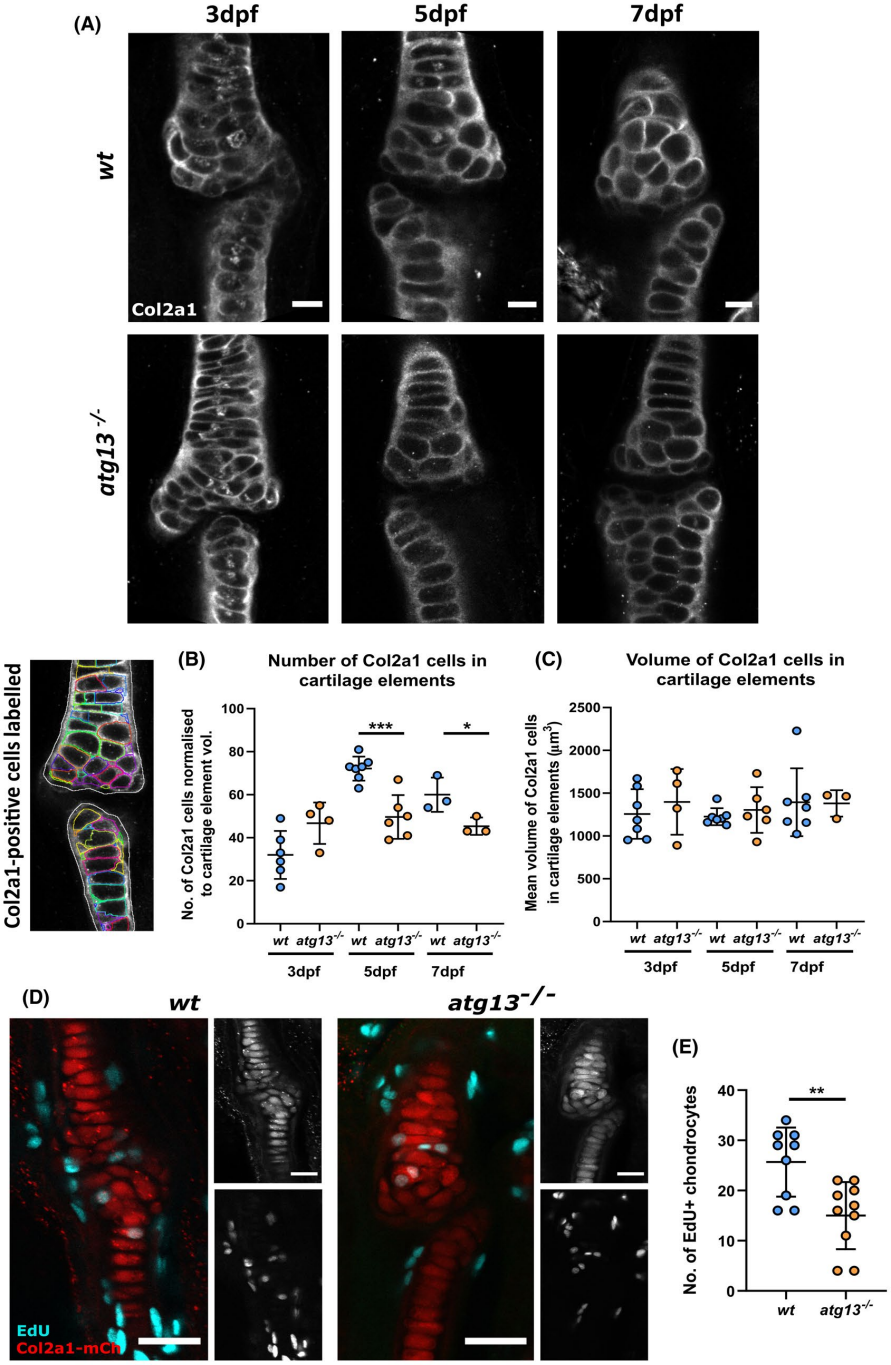
*atg13*‐mutant fish show decreased number of chondrocytes and reduced proliferation at joint site. (A) Representative confocal slices of lower jaw joint at 3, 5 and 7 dpf in *wt* and *atg13*‐mutant fish, immunohistochemically labeled for Col2a1. Scale bars = 20 µm. (B) *Left*, example slice from confocal image showing Col2a1‐positive cells outlined by modular image analysis program run in Fiji. *Right*, quantification of Col2a1‐positive cell number normalized to cartilage element volume, (C) and volume of Col2a1‐positive cells within cartilage element for *wt* and *atg13* mutants. Each data point = one larvae. Student's unpaired *t* test performed where ***p = .0004, *p = .0463. (D) Confocal max projections of larval jaw joint in *Tg(atg13; Col2a1aBAC:mcherry) wt* and *atg13* mutants at 6 dpf following 24‐h treatment with EdU Click‐iT, EdU (cyan) and mCh‐Col2a1 (red). Scale bars = 25 and 20 µm for insets. (E) Quantification of number of EdU positive chondrocytes within jaw joint region (determined as region at 5× zoom on 20× objective, when joint in middle of image plane). EdU positive chondrocytes colocalized to Col2a1 staining and counted by going through *z*‐stack. Student's unpaired *t* test performed, **p = .0032

Using confocal images of larval jaws labelled for Col2a1 at 3, 5, and 7 dpf, the number and volume of all cells from within the cartilage elements forming the joint site was quantified (Figure 5A–C). This was achieved by utilizing a modular image analysis program which can identify and outline individual Col2a1‐positive cells that form the lower jaw joint during development (Figure 5B, *left*). Analysis of the collated data showed that at 5 and 7 dpf, the number of Col2a1‐positive chondrocytes forming the cartilage elements was decreased in the *atg13*‐mutant compared to *wt* when normalized to total element volume (Figure 5B). However, cell volume remained unchanged (Figure 5C). This could indicate decreased cell proliferation as cell volume is unaffected. To investigate this, we performed an EdU proliferation assay on larvae from 5–6 dpf (Figure 5D). By analyzing the number of EdU‐positive chondrocytes at the joint site, we found that the *atg13* mutants showed a decrease in the number of proliferating cells within the joint compared to *wt* (Figure 5E). This implies a role for autophagy in chondrocyte development and maturation. This is of particular significance at joint sites where pre‐chondrogenic cells are beginning to differentiate and mature as they are pushed up the element into the intercalation zone. Therefore, changes to differentiation and maturation here will have the biggest impact upon joint morphology.

### 3.5 Chondrocytes show premature hypertrophication within *atg13* mutants

**FIGURE 6:**
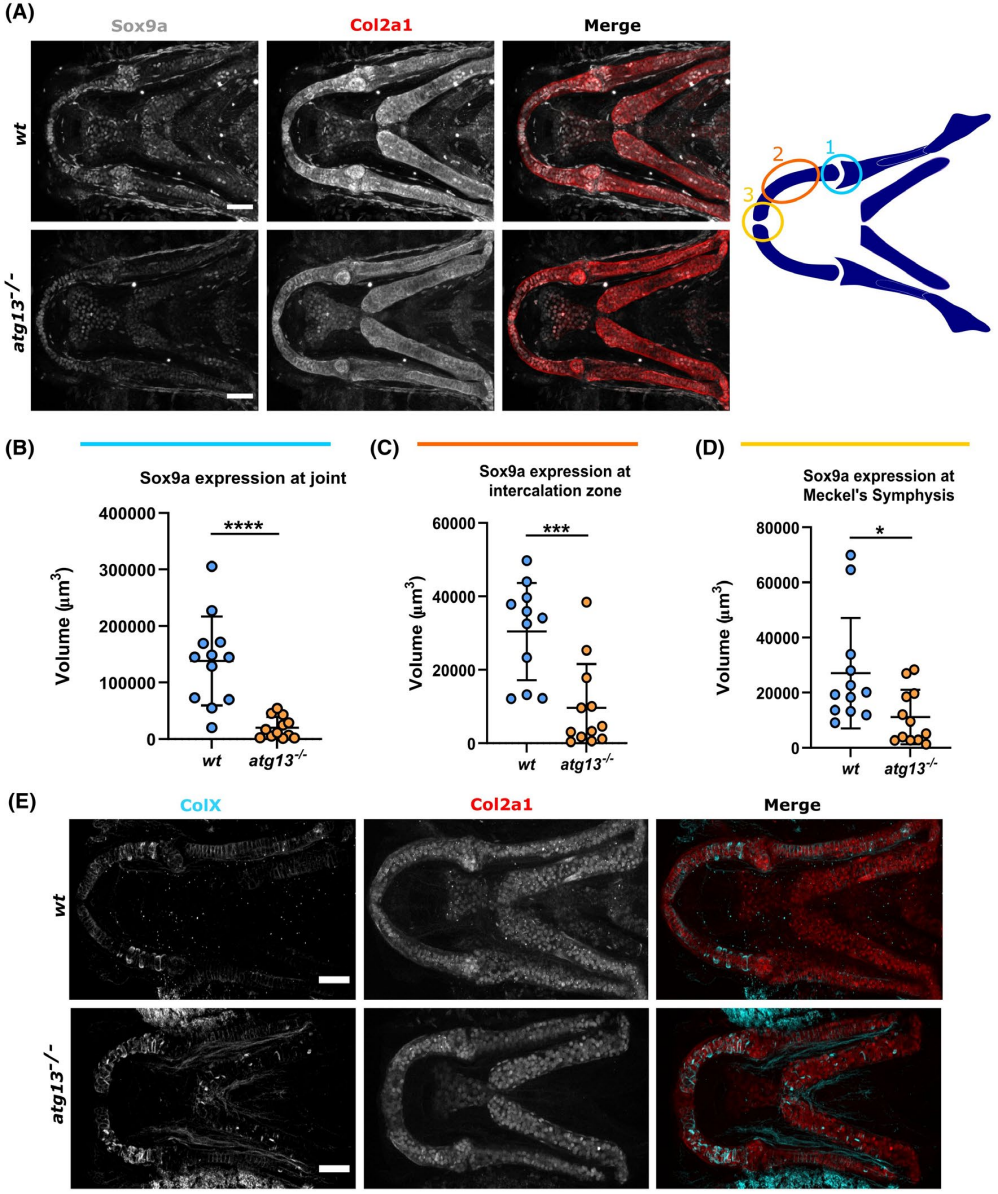
*atg13* mutation affects expression and production of key factors in cartilage development. (A) *Left*, confocal max projections of lower jaw at 5 dpf in *wt* and *atg13*‐mutant fish, immunostained for Sox9a (grey) and Col2a1 (red). Scale bars = 50 µm. *Right*, schematic showing regions of interest selected within lower jaw in modular image analysis program (SoxQuant). Colours correspond to graphs below. (B–D) Quantification of Sox9a expression measured as volume of Sox9a within Col2a1 positive cells from confocal *z*‐stack. Student's unpaired *t* test performed where ****p < .0001, ***p = .0007, *p = .0173; *n* = 12 for both. (E) Confocal max projections of the lower jaw at 7 dpf in *Tg(atg13; Col2a1aBAC:mcherry) wt* and *atg13*‐mutant larvae, immunostained for collagen Type X (ColX) (cyan) and mCherry (for Col2a1, red). Scale bars = 50 µm

SOX9 is one of the first transcription factors expressed within the chondrocyte maturation pathway and is essential for chondrocyte differentiation and subsequent cartilage formation.^73^ The *atg13* mutants show a significant decrease in the expression of Sox9a, an ortholog of tetrapod SOX9,^74^ at three regions within the lower jaw: at the joint site, within the intercalation zone of the Meckel's cartilage, and at the Meckel's cartilage symphysis (Figure 6A–D). Thus, indicating an overall reduction in Sox9a across chondrocytes forming the lower jaw. As chondrocytes mature and become hypertrophic, Sox9a expression decreases, therefore, a reduction in Sox9a expression comparative to *wt* is indicative of premature progression of immature chondrocytes into hypertrophic chondrocytes. To confirm this, we assessed the expression of Col10a1 via immunostaining (Figure 6E). Col10a1 can be used as a marker for hypertrophic chondrocytes, as well as early osteoblasts, as during cartilage maturation chondrocytes switch collagen expression from Col2a1 to Col10a1.^49^ At 7 dpf, we found that the *atg13* mutants had an elevated Col10a1 positive chondrocyte population compared to *wt*, indicating that these cells are maturing more rapidly. Along with reduced Sox9a data, these data indicate that loss of autophagy activity disrupts the rate of chondrocyte maturation and causes chondrocytes to become prematurely hypertrophic.

### 3.6 *atg13*‐mutant chondrocytes show alterations to chondrocyte organization and ECM formation

**FIGURE 7:**
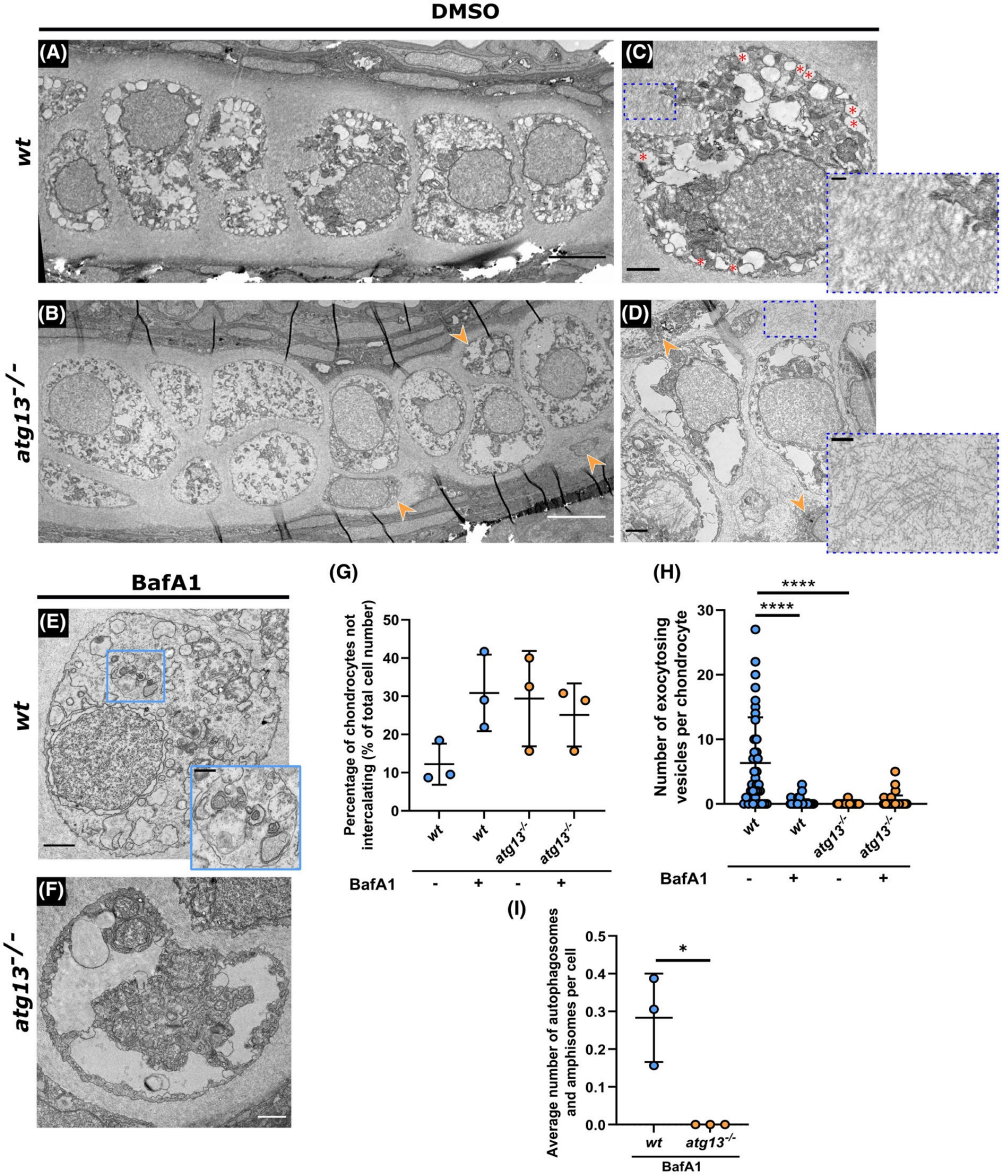
Ultrastructure and organization of chondrocytes affected in *atg13* mutants. (A–D) Electron microscopy of ethmoid plate in *wt* and *atg13*‐mutant fish at 5 dpf following DMSO or (E and F) BafA1 treatment for 3 h. (B and D) Orange arrow heads highlight areas of non‐uniformity and non‐intercalating chondrocytes in *atg13*‐mutant cartilage. (C) Red asterisks show vesicles fusing with outer membrane in *wt*, not present in *atg13* mutant. Blue dotted boxes and inset show differences in ECM organization and density between *wt* and *atg13* mutants. (E and F) BafA1 treatment increases number of vesicles in both *wt* and *atg13* mutants and ablates vesicle‐membrane fusion events. Blue box and inset in (E) shows autophagosome in BafA1 treated *wt* chondrocyte, not present in *atg13* mutants. Scale bars A, B = 5 µm; C–F = 1.5 µm; C’–E’ = 0.5 µm. (G) Number of chondrocytes on periphery of cartilage and not aligning down central line of stack. Calculated as percentage of total cell number along ethmoid plate in one section. *N* = 38 chondrocytes total from 3 larvae, per condition and genotype. (H) Number of vesicles fusing with outer cell membrane quantified per cell following DMSO or BafA1 treatment. Two‐way ANOVA performed for each; ****p < .0001. (I) Average number of autophagosomal structures per chondrocyte in bafilomycin A1 treated fish, calculated as average of all chondrocytes per individual fish. Student's unpaired *t*‐test performed; *p = .0138

To explore how these changes to chondrocyte maturation manifest within the developing tissue, we performed ultrastructural analysis on chondrocytes of the ethmoid plate at 5 dpf (Figure 7). Under basal conditions, *wt* chondrocytes appeared elongated, were arranged in a stacked formation along the length of the cartilage and were surrounded by a dense and organized ECM (Figure 7A). In contrast, the *atg13*‐mutant chondrocytes were more disorganized with increased numbers of immature and non‐ or partially intercalated chondrocytes observed at the cartilage edge, resulting in a non‐uniform and bumpy appearance along on the cartilage border (Figure 7B,D *(orange arrowheads)* and 7G). Compared to *wt*, the *atg13*‐mutant chondrocytes were also less electron dense and larger in size (Figure 7D,I), indicative of late‐stage hypertrophication. These data, together with the changes to sox9a and Col10a1 expression, indicate that the *atg13*‐mutant chondrocytes undergo an altered maturation process which inhibits proper cell placement and intercalation, leading to disorganization of cartilage structure.

Focusing on the intracellular contents of the chondrocytes, in *wt* fish, vesicles containing low‐electron dense material, predicted to be type II collagen fibrils were evident within the cytoplasm, with a substantial population fusing with the plasma membrane during exocytic events (Figure 7C, *red asterisks*). Although similar vesicles could be observed in the *atg13* mutants (Figure 7B,D), very few of those vesicles were found to be undergoing fusion with the plasma membrane (Figure 7H). This suggests that loss of autophagy affects vesicle exocytosis in chondrocytes. Notably, the ECM surrounding the chondrocytes appeared sparser and less well organized in the *atg13* mutants (Figure C and D, *inset*), which we hypothesize is related to reduced vesicle exocytosis, as these vesicles are predicted to contain collagens required for ECM formation.

To explore whether these effects were specific to loss of autophagy caused by the *atg13* mutation, larvae were treated with BafA1 for 3 h at 5 dpf (Figure 7E,F). Similar to the effect of the *atg13* mutation, we found that BafA1 treatment abrogated vesicle exocytosis in *wt* fish (Figure 7H), causing an increase in the number of vesicles within the cytoplasm of *wt* chondrocytes (Figure 7E). This supports the hypothesis that blocking or stalling of the autophagy pathway has a role in vesicle exocytosis within chondrocytes. The *wt* treated larvae also showed an increase in the number of chondrocytes not intercalating (Figure 7G), with more cells present at the outer edges of the cartilage, perpendicular to the main stack, as also seen in the mutants. These data demonstrate a role for autophagy in the control of chondrocyte differentiation and in ECM formation.

## 4 DISCUSSION

In this study, we have explored the role of autophagy in chondrocyte development and maturation. We have shown that loss of a key autophagy protein, Atg13, affects cartilage formation, joint function, and is detrimental to zebrafish larval survival.

Within our model we have found that loss of Atg13 affects early larval development, causing reduced growth and loss of swim bladder inflation, and juvenile lethality by 17 dpf. These data demonstrate that expression of *atg13* is essential for zebrafish survival, and that Atg13 may play both autophagic and non‐autophagic roles in development. This is in line with data from a previous study that showed that loss of Atg13 in zebrafish affects swim bladder inflation and causes larval lethality.^25^ In a murine *Atg13^−/−^* model, Kaizuka et al demonstrated that mutant mice die by embryonic day 17.5 (E17.5) and show growth retardation, as well as myocardial defects.^20^ We see no obvious changes to cardiac development within our zebrafish model and hypothesize that their delayed growth and eventual lethality is in part due to reduced yolk sac metabolism from 1–5 dpf and reduced free feeding beyond 5 dpf due to altered jaw morphology and function. Similarly, Mawed et al. demonstrated that following yolk absorption at 5 dpf, *beclin1* and *atg7* knockout zebrafish mutants were unable to cope with metabolic stress and died at 9 and 15 dpf, respectively.^63^ Defects in hepatic glycogen and lipid metabolism, and within intestinal architecture were also observed in both mutants, indicating that autophagy is critical for energy metabolism during early zebrafish development.

Through its interaction with ULK1 and FIP200, ATG13 is a vital element of the ULK protein kinase complex which is a key signaling node and the first protein complex within the autophagy pathway and is essential for initiating autophagosome formation.^75^, ^76^ Our data show that under basal conditions, the *atg13* mutant has limited autophagy activity, as indicated by a reduction in GFP‐Lc3 puncta compared to *wt*, and an accumulation of p62. As an adaptor protein, p62 helps deliver cargo to the autophagosome by binding to ubiquitinated substrates and LC3, and is mainly degraded by autophagy.^77^ Therefore, an accumulation of p62 indicates autophagy inhibition. The presence of GFP‐Lc3 puncta, although surprising, has been observed in other *atg13* null mouse and *C. elegans* models, and therefore, we predict could be due to LC3 aggregation or activation of a non‐canonical autophagy pathway, such as LAP (Lc3‐associated phagocytosis), where LC3 lipidation can occur independently of the ULK preinitiation complex.^78^, ^79^ Following treatment with Bafilomycin, the *atg13* mutants showed impaired autophagic flux as demonstrated by the minimal increase in GFP‐Lc3 puncta and p62 protein levels. Using electron microscopy, we were also able to detect autophagosomes and amphisomes present in BafA1 treated *wt* chondrocytes which were completely absent in *atg13*‐mutant cells, further demonstrating a loss of autophagic activity caused by the *atg13* mutation (Figure 7E, *blue box inset* and I).

Autophagy is known to be an important pathway in many developmental processes, including early joint and limb formation.^80^, ^81^ Here, autophagy functions in parallel with apoptosis, and other key cellular processes, to help in the remodelling of major tissues as required during embryogenesis.^82^, ^83^ We observe changes to expression of Sox9a in the *atg13* mutants; Sox9 in addition to its role in specification of chondrocytes, has been demonstrated to control entry to chondrocyte hypertrophy, and inactivation of Sox9 in pre‐hypertrophic cells leads to failure to enter hypertrophy and premature apoptosis.^84^ While an investigation of the interplay of cell degenerative processes and autophagy activity was beyond the scope of this current study, it would be interesting to examine the effect that loss of *atg13*, and reduced autophagy flux, has on these co‐ordinated cell death and senescence pathways during joint formation and later during joint homeostasis and ageing.

As mentioned, previous studies using mice have identified roles for autophagy in cartilage development and growth; however, the effect of these changes on joint formation and function has not been discussed. Here we have shown that *atg13*‐mutant zebrafish have reduced jaw function at 5 dpf, which is not caused by alterations to jaw muscle development or gross jaw morphology, but instead correlates with changes to chondrocyte number at the jaw and reduced proliferation of joint precursors. These results are consistent with those from other autophagy‐null murine models which also show decreased chondrocyte proliferation during development, leading to a reduction in long bone length and overall body size from birth to adulthood.^30^, ^50^, ^53^ The *atg13* mutants also showed decreased expression of an early chondrocyte marker, Sox9a. Taken together with the increased expression of Col10a1 in *atg13* mutants, these data suggest that autophagy, and specifically Atg13, has a role in regulating the rate of chondrocyte maturation.

This disruption to chondrocyte maturation was confirmed by ultrastructural analysis as chondrocytes in the *atg13* mutants were more disorganized and had a reduced cellular density. Additionally, in the *atg13* mutants we observed an increase in the number of chondrocytes failing to fully intercalate into the cartilage element; a phenotype which could be induced in *wt* following treatment with BafA1, indicating that these changes may be specifically due to loss of autophagy activity. Sox9 is a key factor in early chondrogenesis and chondrocyte differentiation,^85^ and heterozygous deletion of *Sox9* in mice has been shown to cause premature chondrocyte hypertrophy and accelerated ECM mineralization,^86^ while *sox9* null zebrafish show reduced chondrocyte numbers and absence of proper chondrocyte intercalation.^87^ Meanwhile, in undifferentiated mesenchymal cells, *Sox9* has been shown to be regulated by the serine/threonine protein kinase mTORC1, which is also a master regulator of autophagy.^88^ Therefore, we hypothesize that the effects on chondrocyte maturation and intercalation seen in the *atg13* mutants are due to dysregulation of *sox9a* expression, and that this dysregulation could be mediated by loss of autophagy activity.

Ultrastructural analysis revealed a decrease in ECM density and organization in the *atg13* mutants. This is similar to the ECM phenotype seen in mice with conditional loss of *Atg7* in chondrocytes, which is caused by retention of procollagen 2 within the ER, as demonstrated by the enlarged and highly electron dense ER cisternae in the *Atg7*‐mutant mice.^52^ In our *atg13*‐mutant model, we see no obvious changes to ER structure and distribution, but we do observe a drastic reduction in the number of vesicular exocytosis events. Therefore, we hypothesize that the changes to ECM formation are due to a reduction in the secretion of collagens required for ECM formation via exocytosis. This decrease in collagen secretion could be due to their accelerated maturation, as chondrocytes reduce collagen production as they become more hypertrophic.^89^, ^90^ Alongside this, *Sox9* expression is required for expression of *Col2a1*, along with other collagens,^91^, ^92^ and therefore, its decreased expression in the *atg13* mutant could affect collagen production, leading to reduced secretion and a sparser ECM.

Here, we show that Atg13 has a role in cell differentiation during skeletal development and that changes to autophagy activity have an impact upon how joints are formed and maintained, and function. Given the link between autophagy and OA, our data highlight three possible mechanisms for increased OA risk following loss of Atg13 and dysregulation of autophagy. First, alterations to chondrocyte maturation can lead to altered joint function which can lead to alterations to joint loading throughout life. Second, premature maturation of chondrocytes could lead to increased hypertrophy of articular cartilage, leading to mineralization, and thirdly, reduced cartilage matrix secretion could lead to cartilage that is less able to withstand physiological load and is at higher risk of breakdown. Therefore, our results identify potential links between specific autophagy proteins and cartilage health which can be used to improve our understanding of joint diseases such as OA.

## DISCLOSURES

The authors declare no conflicts of interest with this article.

## AUTHOR CONTRIBUTIONS

Joanna J. Moss, Martina Wirth, Sharon A. Tooze, Jon D. Lane, and Chrissy L. Hammond conceptualized the study and designed experiments. Joanna J. Moss and Martina Wirth conducted the experiments and interpreted data. Joanna J. Moss performed statistical analysis and made all figures. Joanna J. Moss, Jon D. Lane, and Chrissy L. Hammond wrote the first draft of the manuscript and all authors made intellectual contributions and assisted in manuscript editing.

## Supporting information

Fig S1‐S4

Video S1

Video S2
